# Population-based cohort study of modifiable risk factors for dementia and Alzheimer disease mortality in Spain

**DOI:** 10.1038/s44528-026-00022-5

**Published:** 2026-09-02

**Authors:** Julián Benito-León, José Ignacio Serrano, María Dolores del Castillo, Álex Escolà-Gascón, Carla María Benito-Rodríguez, Cristina Ramo-Tello, Ritwik Ghosh, Félix Bermejo-Pareja

**Affiliations:** 1 Department of Neurology, 12 de Octubre University Hospital, Madrid, Spain.; 2 Group of Neurodegenerative Diseases, Hospital Universitario 12 de Octubre Research Institute (Imas12), Madrid, Spain.; 3 Network Center for Biomedical Research in Neurodegenerative Diseases (CIBERNED), Madrid, Spain.; 4 Department of Medicine, Faculty of Medicine, Complutense University of Madrid, Madrid, Spain.; 5 Computational Models of Intelligence group, Center for Automation and Robotics (CAR, CSIC-UPM), Consejo Superior de Investigaciones Científicas (CSIC), Arganda del Rey, Spain.; 6 STREAM Research Group, Ramon Llull University, Barcelona, Spain.; 7 Department of Neurology, Torrejón University Hospital, Torrejón de Ardoz, Madrid, Spain.; 8 Department of General Medicine, Burdwan Medical College & Hospital, Burdwan, West Bengal, India.

## Abstract

**Background:**

Although many modifiable factors are linked to dementia risk, their association with dementia and Alzheimer disease (AD) mortality remains unclear. We aimed to identify late-life modifiable factors associated with these outcomes and assess sex differences.

**Methods:**

In the population-based Neurological Disorders in Central Spain (NEDICES) cohort, we followed 3085 older adults enrolled in 1994–1995 through 2017. We examined 13 modifiable factors measured at study entry. Dementia and AD deaths were defined by the underlying cause recorded on death certificates. Fine–Gray competing risk models accounted for death from other causes and generated subdistribution hazard ratio (SHR) estimates and adjusted population attributable fractions (PAFs).

**Results:**

During follow-up, there were 182 dementia-coded deaths (including 105 AD-coded deaths) and 2423 deaths from other causes. Here we show that depressive symptoms are associated with higher dementia-coded mortality in women (SHR 1.52, 95% confidence interval [CI] 1.00–2.30; PAF 13.6%, 95% CI 0.0–28.4) and higher AD-coded mortality overall (SHR 1.76, 95% CI 1.11–2.79; PAF 15.4%, 95% CI 2.6–29.9), with a stronger association in women (SHR 2.23, 95% CI 1.28–3.88; PAF 27.2%, 95% CI 7.8–46.7). Several exposures show inverse associations (e.g., smoking, obesity, and arterial hypertension in men), consistent with competing mortality and late-life reverse causation. Combined positive PAFs are 10.8% for dementia-coded mortality and 18.2% for AD-coded mortality overall, reaching 41.1% in women for AD-coded mortality.

**Conclusions:**

Late-life depressive symptoms, particularly among women, emerged as the most prominent modifiable correlate of dementia- and AD-coded mortality, supporting improved recognition and management of depressive symptoms in older adults.

Dementia is a major and growing contributor to mortality and disability worldwide. In 2019, it was the seventh leading cause of death globally; Alzheimer disease (AD) and other dementias accounted for ~1.62 million deaths, a burden expected to rise as populations age^1,2^. An analysis of 2019 claims from Medicare—the U.S. federal health insurance program—showed that approximately one in three beneficiaries who died that year had previously received a diagnosis of AD or another form of dementia, underscoring both increasing prevalence and the ultimately fatal trajectory of these disorders^3^. Yet dementia is frequently under-recorded on death certificates, leading to systematic underestimation of dementia-related mortality and delayed public health action^4–6^. Together, these realities underscore the urgency of identifying and mitigating modifiable risk factors that can prevent or delay dementia onset and, downstream, reduce dementia-related deaths^1–7^.

A substantial fraction of dementia risk is attributable to modifiable exposures across the life course. These modifiable exposures include lower educational attainment, hearing impairment, depression, traumatic brain injury, physical inactivity, diabetes mellitus, smoking, arterial hypertension, obesity, excessive alcohol consumption, social isolation, air pollution, visual impairment, and elevated LDL cholesterol^8^. Not all are available in every cohort; therefore, studies often operationalize a subset.

The 2024 Lancet Standing Commission expanded its prevention framework from 12 to 14 risk factors and estimated that approximately 45% of dementias could be prevented or delayed if these exposures were addressed; the update added untreated vision impairment (late life) and high LDL-cholesterol (midlife)^8^. This extends earlier Lancet Commission estimates (~40%) and reinforces prevention as a central strategy in the setting of limited curative options for established disease^9,10^. Earlier population-attributable-risk modeling estimated that up to approximately half of AD cases worldwide might be statistically attributable to seven potentially modifiable factors: midlife hypertension, diabetes, smoking, depression, physical inactivity, midlife obesity, and low educational attainment^11^.

A critical gap remains: to what extent do modifiable factors measured in late life translate into the long-term probability of dying from dementia, particularly when dementia (or AD) must be recorded as the underlying cause of death? Most epidemiologic evidence focuses on incident dementia; far fewer studies have evaluated dementia-specific mortality over long follow-up^12–16^. Mortality analyses are also vulnerable to competing risks, because many older adults die from other causes before dementia is recorded as the underlying cause of death^17–19^. Risk factors that increase overall mortality may appear inversely associated with dementia death because exposed individuals die earlier from non-dementia causes, a form of survival/competing-risk bias that has been invoked for exposures such as smoking or late-life obesity^17–19^. Addressing this challenge requires prolonged observation and explicit competing-risk methods that estimate cumulative incidence while accounting for deaths from other causes^20–22^.

Few longitudinal studies have evaluated a broad panel of modifiable factors in relation to both dementia-specific and AD-specific mortality across decades while explicitly accounting for competing risks. In the Neurological Disorders in Central Spain (NEDICES) cohort, dementia contributes substantially to all-cause mortality over 13 years, and selected exposures (e.g., long sleep duration) have been linked to dementia-specific death; complementary work in mild cognitive impairment has described cause-specific mortality patterns^16,23,24^. However, these analyses did not jointly evaluate multiple modifiable risks within a competing-risk framework for dementia- and AD-coded death^16,23,24^.

Here, we analyze 23-year mortality outcomes in NEDICES, a large community-based cohort of older adults with detailed study-entry assessment of health and lifestyle factors^25–27^. We use Fine–Gray subdistribution hazard models to estimate associations between each modifiable factor and the cumulative incidence of dementia-specific and AD-specific death while accounting for the competing risk of death from other causes, in line with best-practice guidance^20–22^. To quantify the potential population-level contribution of the examined factors to dementia- and Alzheimer disease–coded mortality, we calculated adjusted population-attributable fractions (PAFs) and combined PAFs accounting for overlap among risk factors, using established methods^11^.

We hypothesize that modifiable factors prominent in incidence research (e.g., physical inactivity, diabetes, depression, and social isolation) are associated with long-term dementia-specific and AD-specific mortality, and that associations may differ by sex, given longer female survival and higher late-life dementia burden versus historically greater male exposure to certain risks (e.g., smoking)^1,3^. By conducting separate competing-risk analyses for dementia-coded death and AD-coded death, and stratifying results by sex, we aim to inform life-course prevention strategies that may reduce both dementia incidence and dementia-related mortality^8–11^.

## Methods

### Standard protocol approvals, registrations, and patient consents

All procedures involving human participants were approved by the Clinical Research Ethics Committees of 12 de Octubre University Hospital and La Princesa University Hospital, both in Madrid, Spain. Written informed consent was obtained from all participants.

### Study population

Data for the present analyses were derived from the Neurological Disorders in Central Spain (NEDICES) study, a population-based investigation of the prevalence, incidence, and determinants of age-related neurological and chronic conditions in older adults. The study was conducted in three socioeconomically and geographically distinct regions of central Spain: two urban neighborhoods—Lista (a middle- to high-income district in Madrid) and Las Margaritas (a lower-income area in Getafe)—and one rural area, the county of Arévalo in the province of Ávila. Detailed descriptions of sampling, diagnostic procedures, and cohort characteristics have been published elsewhere^25–27^.

All participants were aged ≥65 years at enrollment; therefore, exposures were assessed at study entry in late life. For the present analyses, we restricted the sample to participants who were dementia-free at study entry (1994–1995) and excluded those with prevalent dementia. Because AD at baseline was defined as an etiologic subtype of dementia (NINCDS ADRDA criteria)^28^, prevalent AD dementia was captured within these exclusions, and a separate exclusion for prevalent AD was not required. Although NEDICES included follow-up assessments (including a second wave in 1997–1998), we defined exposures using only the 1994–1995 study-entry assessment and did not model time-updated covariates. Mortality outcomes were ascertained through linkage to national vital statistics through 31 December 2017 (see below).

### Study evaluation

At study entry (1994–1995), participants completed a structured questionnaire assessing sociodemographic characteristics, education, medical and medication history, and lifestyle variables, including smoking status (current smoker vs non-current [ex-smoker or never]) and alcohol consumption (current drinker vs non-current [ex-drinker or never])^25–27^. Because the available study-entry coding combined former and never users, we modeled smoking and alcohol as binary indicators (current vs non-current); former and never users could not be separated in the primary analyses. Additional items captured social activities and sensory function (self-reported hearing or vision impairment). Sleep duration was obtained by summing the reported hours of nighttime sleep and daytime naps, as previously described^16,29–31^.

Physical activity (sedentary vs active) was assessed at study entry using an adapted, four-item version of the Rosow–Breslau physical function measure^32^, as previously applied in NEDICES^33,34^. Trained interviewers asked participants to report the daily hours they dedicated to: (a) sedentary lifestyle (minimal household chores or only short walks at home); (b) light activity (regular household chores and independent walking at home); (c) moderate activity (regular household chores and walking up to approximately 1 km/day); and (d) high activity (heavy housework, walking >1 km/day, or regular sports). For the present analyses, we dichotomized physical activity into sedentary (category a) versus active (any light/moderate/high activity; categories b–d)^33,34^. Because the instrument is based on self-reported daily time across these categories, no predefined weekly minute thresholds were applied.

A screening question for depressive symptoms was administered (‘Do you suffer from depression?’), which we used to define depressive symptoms (yes/no) at study entry. The question was modeled on that used in a previous study^35^, in which it correctly identified depression in 85.4% of participants and performed comparably to the 30-item Geriatric Depression Scale. Within the NEDICES cohort, this question was further validated in a subsample of 50 participants, showing 86% concordance (κ = 0.72, sensitivity = 90.5%, specificity = 82.8%), with similar accuracy among those with and without neurological disorders^36^. This validation supports its use as a reliable indicator of clinically significant depressive symptoms in population-based studies^16,36–38^. Accordingly, throughout the manuscript, we refer to this exposure as depressive symptoms rather than a formal clinical diagnosis of major depressive disorder.

Dementia was diagnosed according to the Diagnostic and Statistical Manual of Mental Disorders, Fourth Edition (DSM-IV) diagnostic criteria and adjudicated by neurologists experienced in dementia diagnosis^39–41^. AD (including both possible or probable categories) was diagnosed using NINCDS ADRDA criteria^28^.

### Mortality data

Vital status and causes of death were ascertained through December 31, 2017, via linkage to the Spanish National Population Register (*Instituto Nacional de Estadística*). Follow-up ended on December 31, 2017, as this was the latest date with complete, validated linkage to national mortality records and coded underlying causes of death for the NEDICES cohort. More recent years were not included to preserve uniform outcome ascertainment. Because ascertainment relied on national registry linkage, participants were not lost to follow-up for vital status, except for those with unreliable mortality linkage who were excluded (*n* = 52, see below). Participants alive on December 31, 2017, were treated as right-censored at that date, contributing person-time up to that date. Causes of death were coded according to the International Classification of Diseases, 10th Revision (ICD-10). We defined two mortality endpoints based on the underlying cause of death recorded on the death certificate: (1) dementia-coded mortality, defined as ICD-10 F01–F03 or G30, and (2) AD-coded mortality, defined as ICD-10 G30. These endpoints were analyzed in separate competing-risk models, each treating death from other causes as the competing event. For deaths coded as dementia (F01–F03 or G30), we reviewed available NEDICES clinical records, when present, to support subtype classification; however, endpoint assignment for the primary analyses was based on the ICD-10 underlying cause code.

### Study-entry risk (exposure) factors

We examined 13 modifiable study-entry exposures selected a priori based on established dementia-prevention literature and their availability in the NEDICES cohort, and coded them as binary (present/absent) unless otherwise stated.

The 13 study-entry modifiable exposures included diabetes mellitus (self-reported physician diagnosis), arterial hypertension (self-reported history of arterial hypertension), hypercholesterolemia (self-reported diagnosis), current smoking (yes/no), alcohol consumption (current drinker vs non-current [ex-drinker or never]), physical inactivity (sedentary vs any light/moderate/high activity), depressive symptoms (significant symptoms on screening), living alone (solitary household; proxy for social isolation), hearing impairment (self-reported moderate or severe difficulty with or without hearing aid), visual impairment (self-reported moderate or severe difficulty even with correction), sleep duration (<6 or >8 h vs 6–8 h, derived from reported nighttime sleep plus daytime napping), obesity (body mass index ≥30 kg/m^2^, computed from height and weight obtained at study entry), and place of residence (urban vs rural). Educational level (primary or less, including illiteracy or read–write only, versus secondary or higher) was evaluated as a candidate factor but was not included in the primary multivariable models used for PAF estimation due to multicollinearity with other sociodemographic covariates (see below).

Because sleep was measured as total daily sleep time rather than nighttime sleep alone, we did not pre-specify additional extreme cut-points (e.g., >10 h/night), and statistical power to examine rare extremes was limited. Place of residence was determined by recruitment site (urban areas: Lista and Las Margaritas; rural area: Arévalo county) and used as a proxy for urbanicity and related environmental context. Because individual-level air-pollution estimates were not available, this variable captures a composite of urbanicity-related factors and should not be interpreted as a direct measure of air-pollution exposure.

### Statistical analyses

Differences in sociodemographic characteristics between the selected and excluded cohorts were assessed using two-sided independent-samples t tests for continuous variables, after evaluation of normality with the Shapiro–Wilk test and Q–Q plots, and two-sided Pearson chi-square tests for categorical variables. *P* values from these primary tests were asymptotic, two-sided, and unadjusted. For the multi-category educational-attainment comparison, Bonferroni-adjusted post hoc pairwise comparisons were performed.

To model dementia- and AD-specific mortality while accounting for death from other causes as a competing event, we fitted Fine–Gray subdistribution hazard models and report subdistribution hazard ratios (SHRs) derived from the cumulative incidence function (CIF), following Austin and Fine^20^. Models were estimated in STATA/SE v17.0 (StataCorp LLC; TX, USA) using the stcrreg command. Models were specified a priori and, in the full cohort, adjusted for age (continuous) and sex; sex-stratified analyses were adjusted for age. All models included mutual adjustment for the study-entry modifiable factors. History of cardiovascular/cerebrovascular disease at study entry (e.g., prior stroke, myocardial infarction, or coronary heart disease) was excluded because these established disease states may arise downstream of upstream vascular risk factors, and conditioning on them would shift the estimand away from the population impact of upstream modifiable exposures.

Multicollinearity among candidate covariates was assessed using variance inflation factors (VIFs) and was considered potentially problematic when VIF > 5. Educational level exceeded this threshold, largely due to collinearity with urban/rural residence, and was therefore excluded from the primary models. As a sensitivity analysis, we retained education as an exposure while removing urban residence.

Adjusted PAFs for each risk factor were computed from the SHR and exposure prevalence (P_e_) in the analytic sample using Equation 1:

(Equation 1) $$\text{Adjusted}\;PAF_{j}=\frac{P_{e}\times \left(SHR_{j}-1\right)}{P_{e}\times \left(SHR_{j}-1\right)+1}.$$

Ninety-five percent confidence intervals (CIs) for each PAF were obtained by applying the same transformation to the lower and upper 95% CI limits of the SHR while holding P_e_ constant.

To summarize the joint population impact of modifiable exposures associated with higher dementia/AD-coded mortality, we calculated combined positive PAFs using only risk-increasing factors (i.e., individual adjusted PAF_j_ > 0). The simple combined positive PAF followed Barnes and Yaffe^11^ (Equation 2):

(Equation 2) $$\text{Combined}\;\text{positive}\;\text{PAF}=1-\prod_{j=1}^{k}\left(1-\text{AdjustedPA}F_{j}\right).$$

All positive PAFs were included regardless of individual statistical significance; uncertainty should be interpreted using the corresponding 95% CIs for the component estimates.

We also calculated a communality-weighted combined positive PAF using the approach of Norton et al.^42^. Communalities (h_j_^2^) were obtained from principal component analysis of the tetrachoric/polychoric correlation matrix of the dichotomous risk factors, and weights were defined as:

$$w_{j}=1-h_{j}^{2}.$$

We then defined a weighted PAF for each factor as:

$$\text{Weighted}\;PAF_{j}=w_{j}\times \text{Adjusted}\;PAF_{j},$$

and computed the weighted combined positive PAF (Equation 3):

(Equation 3) $$\text{Weighted}\;\text{combined}\;\text{positive}\;\text{PAF}=1-\prod_{j=1}^{k}\left(1-\right.\text{Weighted}\;\left.PAF_{j}\right).$$

## Results

### Final selection of study participants

**Fig. 1 |:**
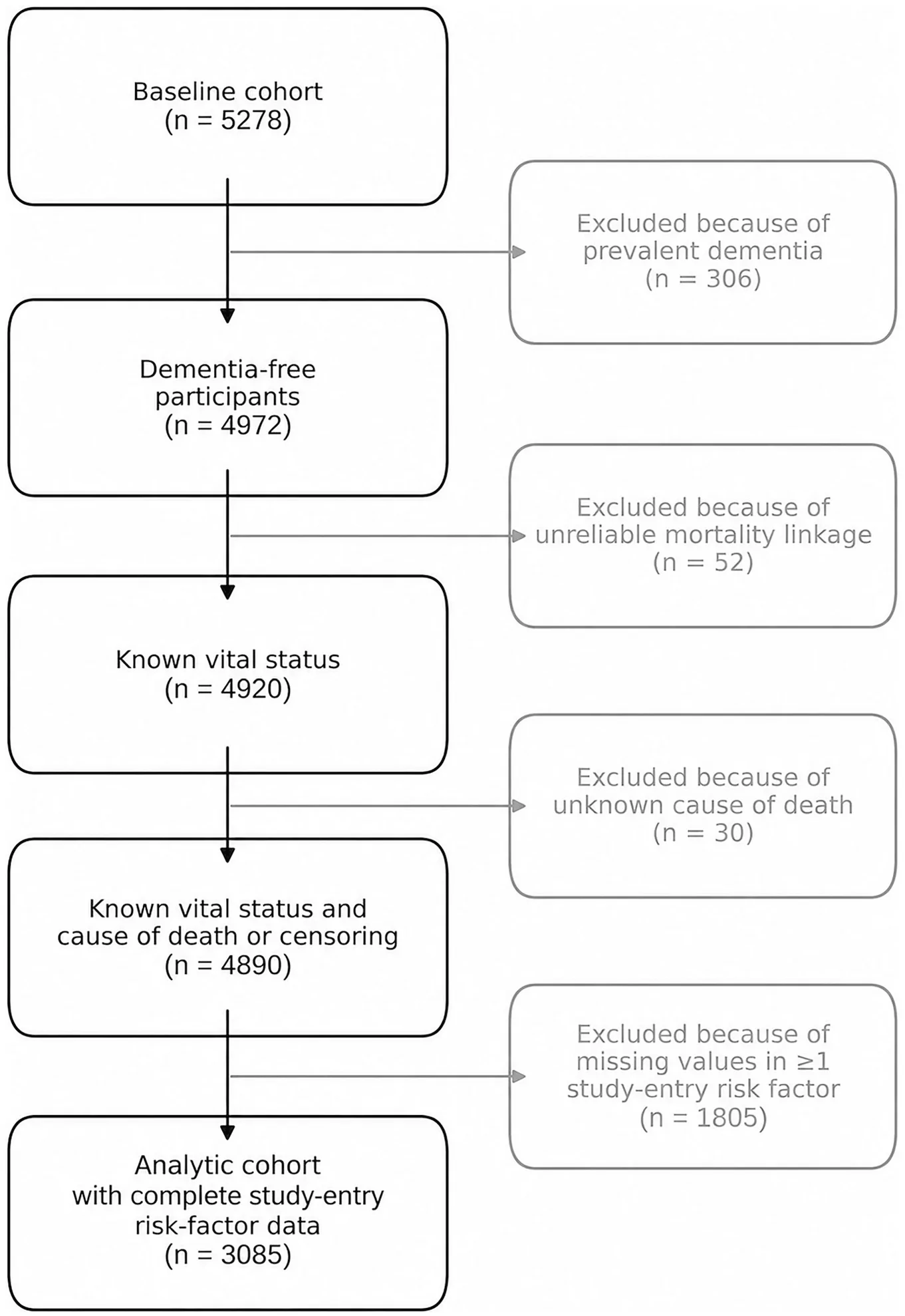
Participant selection. Flow diagram of the NEDICES analytic cohort. Of 5278 participants enrolled at study entry (1994–1995), 306 with prevalent dementia were excluded. Participants were further excluded due to unreliable mortality data (*n* = 52), unknown cause of death (*n* = 30), and missing values in ≥1 study-entry risk factor (*n* = 1805), leaving 3,085 dementia-free participants with complete exposure data and known vital status through 31 December 2017.

Of the 5278 participants initially enrolled at study entry, 306 with prevalent dementia were excluded, yielding 4972 dementia-free individuals. Of these, 52 were further excluded due to unreliable mortality data, and 30 due to unknown cause of death, leaving 4890 participants with known vital status and cause of death or censoring. Participants who were alive at the administrative censoring date (31 December 2017) were retained and treated as right-censored (i.e., they contributed follow-up time up to that date). An additional 1805 participants were excluded due to missing values in one or more study-entry risk-factor variables. The final analytic cohort thus comprised 3085 dementia-free participants with complete exposure data and known death status or censoring through 31 December 2017 (Fig. 1).

Compared with the 1887 dementia-free individuals who were excluded (missing data, unknown cause of death, or unreliable mortality information), the 3,085 included participants were significantly younger on average (mean age 73.4 ± 6.3 vs 74.5 ± 7.0 years; t(4970) = 5.752, *p* < 0.001) and less frequently female (53.8% vs 62.1%; χ^2^(1) = 33.158, *p* < 0.001). Educational attainment also differed (χ^2^(3) = 57.803, *p* < 0.001): those included were less often illiterate (9.8% vs 17.0% of excluded) and more likely to have completed primary education (35.1% vs 31.0%) or secondary/higher education (14.4% vs 12.8%). Bonferroni-corrected pairwise comparisons confirmed that differences in illiteracy and primary education remained statistically significant (both *p* < 0.05).

### Cohort characteristics

Over the 23-year follow-up, there were 182 dementia-coded deaths (5.9%; underlying cause ICD-10 F01–F03 or G30) and 105 AD-coded deaths (underlying cause ICD-10 G30). Because ICD-10 G30 is included within the dementia-coded definition, AD-coded deaths contribute to the overall count of dementia-coded deaths; nevertheless, dementia-coded and AD-coded mortality were modeled as separate endpoints in distinct competing-risk analyses. In addition, 2423 participants (78.6%) died from other causes (competing events), and 480 (15.6%) were alive (censored) at the end of follow-up (31 December 2017). The mean age at study entry was 73.4 years (standard deviation [SD] 6.3), and 53.8% were women. Study-entry characteristics are summarized in Table 1. The mean overall follow-up time was 13.48 years (SD 7.26). For women, it was 14.73 (SD 7.09) while for men it was 12.02 (SD 7.19). Women were slightly older than men (73.6 ± 6.5 vs 73.1 ± 6.2 years; *p* = 0.046) and had lower educational attainment (low education: 89.0% vs 81.6%; *p* < 0.001). Women had a higher prevalence of metabolic factors (arterial hypertension, hypercholesterolemia, obesity; all *p* < 0.001), whereas current smoking (21.9% vs 3.4%; *p* < 0.001) and alcohol consumption (50.6% vs 20.1%; *p* < 0.001) were more prevalent in men. Depressive symptoms (30.4% vs 16.0%; *p* < 0.001) and living alone (24.4% vs 6.7%; *p* < 0.001) were more common in women. Urban residence, hearing impairment, and sleep duration outside 6–8 h did not differ significantly by sex (Table 1).

### Risk factors for dementia-related mortality

We examined 13 modifiable study-entry risk factors in relation to dementia-specific mortality (dementia listed as the underlying cause of death). Fine–Gray competing-risk regression yielded SHRs for each factor (adjusted for the other factors), and Table 2 reports SHRs alongside adjusted PAFs and their 95% CIs.

In the overall cohort, depressive symptoms showed the largest risk-increasing association with dementia-coded death (SHR = 1.414, 95% CI 0.985–2.030). Several factors showed inverse associations (SHR < 1), including current smoking (SHR = 0.614, 95% CI 0.412–0.914) and obesity (SHR = 0.566, 95% CI 0.376–0.852), whereas other factors were imprecisely estimated (Table 2).

Sex-stratified analyses indicated distinct patterns. Among men, arterial hypertension was strongly inversely associated with dementia-coded death (SHR = 0.296, 95% CI 0.140–0.628). No factor showed a statistically significant risk-increasing association in men; however, several factors had SHRs >1 (e.g., diabetes, physical inactivity, hearing impairment, alcohol consumption) with wide confidence intervals (Table 2). Among women, depressive symptoms were associated with a higher subdistribution hazard of dementia-coded death (SHR = 1.519, 95% CI 1.001–2.304), while obesity was inversely associated (SHR = 0.496, 95% CI 0.309–0.796).

Adjusted PAFs were derived from SHRs and exposure prevalence; PAF 95% CIs are shown in Table 2. In the overall cohort, depressive symptoms had a positive PAF of 9.0% (95% CI − 0.4 to 19.7), whereas current smoking and obesity had negative PAFs whose CIs excluded 0 (smoking −4.9%, 95% CI − 7.6 to −1.0; obesity −13.6%, 95% CI: −20.8 to −4.3), reflecting their inverse SHRs. In men, the inverse association with arterial hypertension translated into a large negative PAF (− 44.9%; 95% CI, −60.9 to −19.6). In women, depressive symptoms produced the largest positive PAF (13.6%, 95% CI 0.0 to 28.4), while obesity had a negative PAF (−19.7%, 95% CI − 29.2 to −7.1). Negative PAFs indicate inverse associations with dementia-coded death in a competing-risk framework and should not be interpreted as biological “protection.”

**Fig. 2 |:**
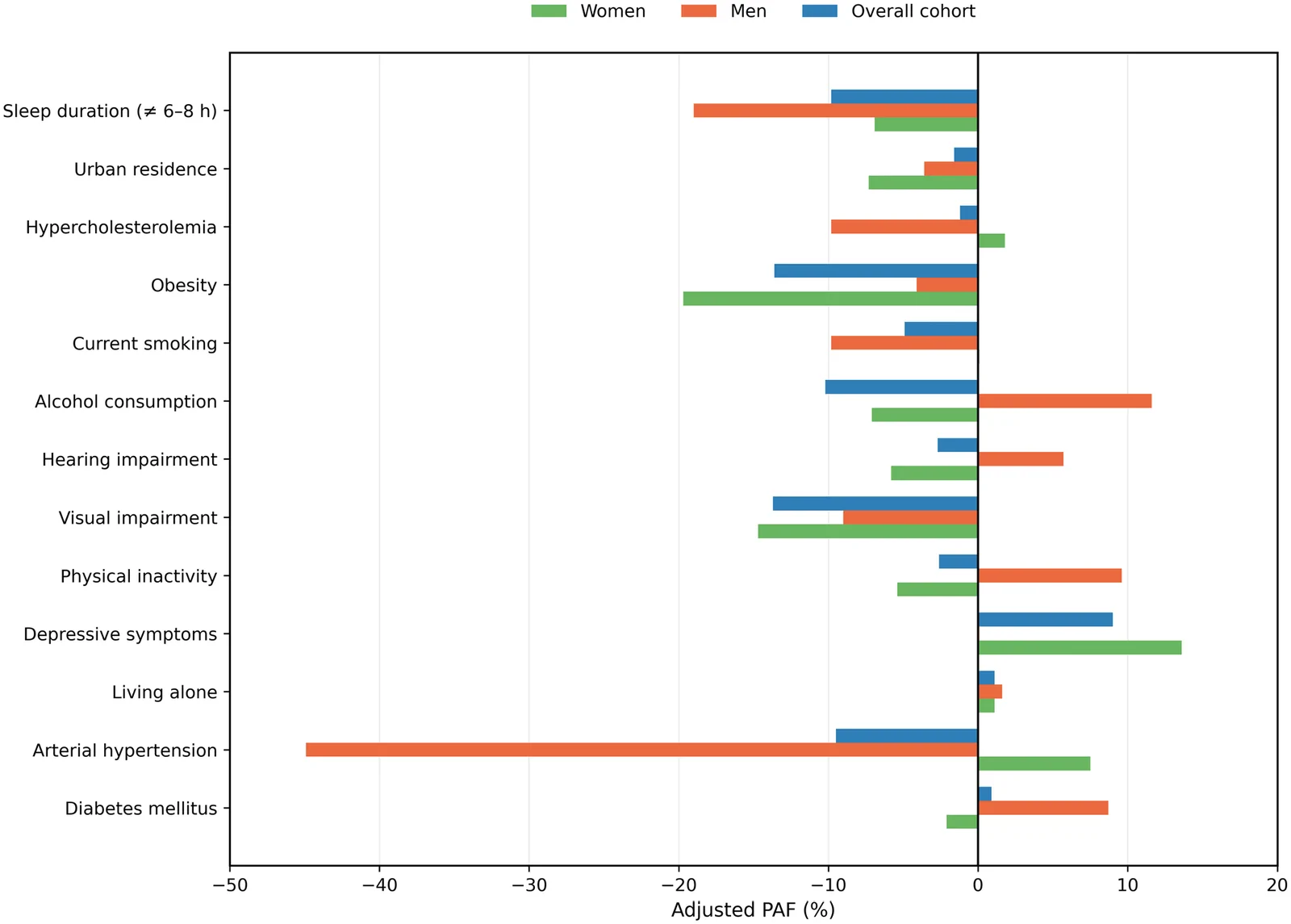
Adjusted population attributable fractions for dementia-coded mortality. Bars show adjusted population attributable fractions (PAFs; point estimates) for dementia-coded death (underlying cause of death) from multivariable Fine–Gray models with death from other causes as a competing event, stratified by sex and shown for the overall cohort. Positive values indicate risk-increasing associations; negative values indicate inverse associations in this competing-risk mortality framework. PAF 95% confidence intervals are reported in Table 2.

**Fig. 3 |:**
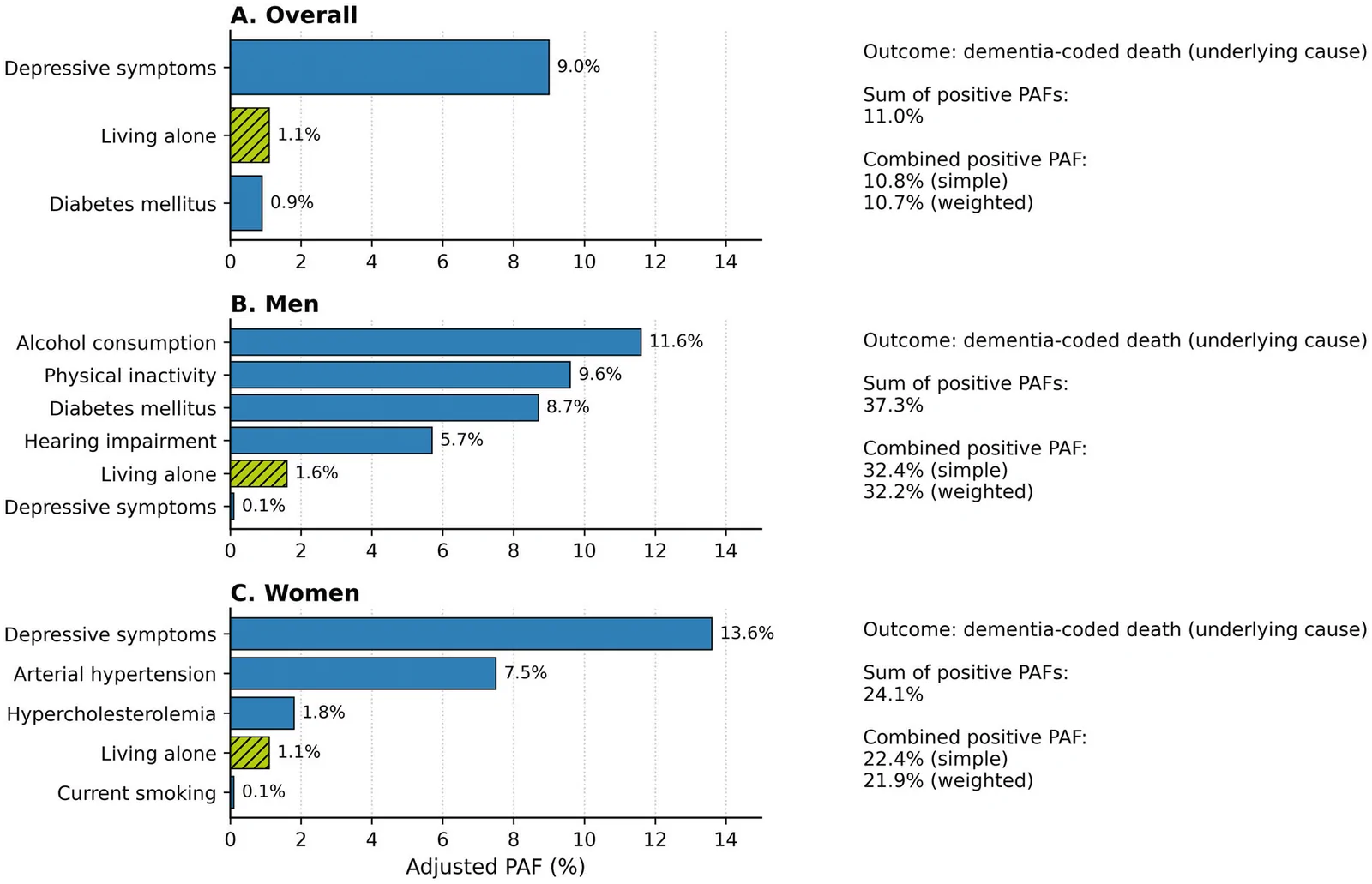
Positive population attributable fractions (PAFs) for dementia-coded mortality and combined positive PAFs by sex. Panels show **A** the overall cohort, **B** men, and **C** women. Horizontal bars display adjusted PAFs (%) for modifiable study-entry factors with positive PAFs only (i.e., factors associated with a higher cumulative incidence of dementia-coded death as the underlying cause of death) estimated from multivariable Fine–Gray competing-risk models with death from other causes treated as the competing event. Bars are ordered from largest to smallest within each panel. The psychosocial factor “living alone” (a proxy for social isolation) is highlighted in green to distinguish it from the remaining factors (shown in the standard color), which represent cardiometabolic/behavioral/health-related exposures. A summary box in each panel reports: (i) the sum of positive PAFs (ΣPAF^+^) and (ii) the combined positive PAF (simple and communality-weighted). Importantly, the combined positive PAF is not the arithmetic sum of the individual bars. It is calculated to avoid double-counting overlap among exposures as: $\text{Combined}\;\text{positive}\;\text{PAF}=1-\prod_{j=1}^{k}\;\left(1-\right.\text{Adjusted}\left.{\text{PAF}}_{j}\right)$. where the product is taken only over factors with positive PAFs in that panel. Individual PAF 95% confidence intervals are reported in Table 2.

Figures 2 and 3A present the adjusted PAFs by factor (overall and by sex). When considering only risk-increasing factors jointly (i.e., combined PAFs based on positive individual PAFs), the combined PAF for dementia-coded death was 10.8% in the total cohort (weighted 10.7%). The combined PAF was higher in men (32.4%; weighted 32.2%) than in women (22.4%; weighted 21.9%) (Fig. 3B, C). Weighted and unweighted combined PAFs were nearly identical because communalities for the included risk-increasing factors were low (Table 2).

In sensitivity analyses, we retained low education (low vs higher educational attainment) and removed urban residence from the mutually adjusted models (Table 4). Among women, depressive symptoms remained a risk-increasing factor for dementia-coded mortality, although the estimate was attenuated and less precise (SHR = 1.183, 95% CI 0.803–1.742; adjusted PAF = 5.3%, 95% CI −6.4 to 18.4).

### Risk factors for Alzheimer disease–specific mortality

We repeated the competing-risk analyses using AD-coded death (ICD-10 G30 as the underlying cause of death) as a separate endpoint; Table 3 summarizes SHRs and adjusted PAFs with 95% CIs.

In the overall cohort, depressive symptoms were associated with higher AD-coded mortality (SHR = 1.763, 95% CI 1.114–2.791), corresponding to a positive PAF of 15.4% (95% CI 2.6–29.9). Visual impairment were inversely associated with AD-coded mortality (SHR = 0.614, 95% CI 0.398–0.947), with a negative PAF of −26.3% (95% CI −48.0 to −2.9). Obesity also showed an inverse association (SHR = 0.579, 95% CI 0.345–0.974), corresponding to a negative PAF of −13.1% (95% CI −22.1 to −0.7). Other factors were not clearly associated with AD-coded mortality in the overall cohort (Table 3).

In sex-stratified analyses, the association between depressive symptoms and AD-coded mortality was concentrated in women: SHR = 2.228 (95% CI 1.278–3.883), PAF = 27.2% (95% CI 7.8–46.7). In women, visual impairment (SHR = 0.536, 95% CI 0.300–0.956) and obesity (SHR = 0.517, 95% CI 0.276–0.967) were inversely associated with AD-coded mortality, with negative PAFs of −36.2% (95% CI −67.0 to −2.6) and −18.8% (95% CI −31.0 to −1.1), respectively. In men, no factor reached statistical significance, although arterial hypertension showed an inverse trend, with an upper CI bound at 1.000 (SHR = 0.464, 95% CI 0.215–1.000) (Table 3).

**Fig. 4 |:**
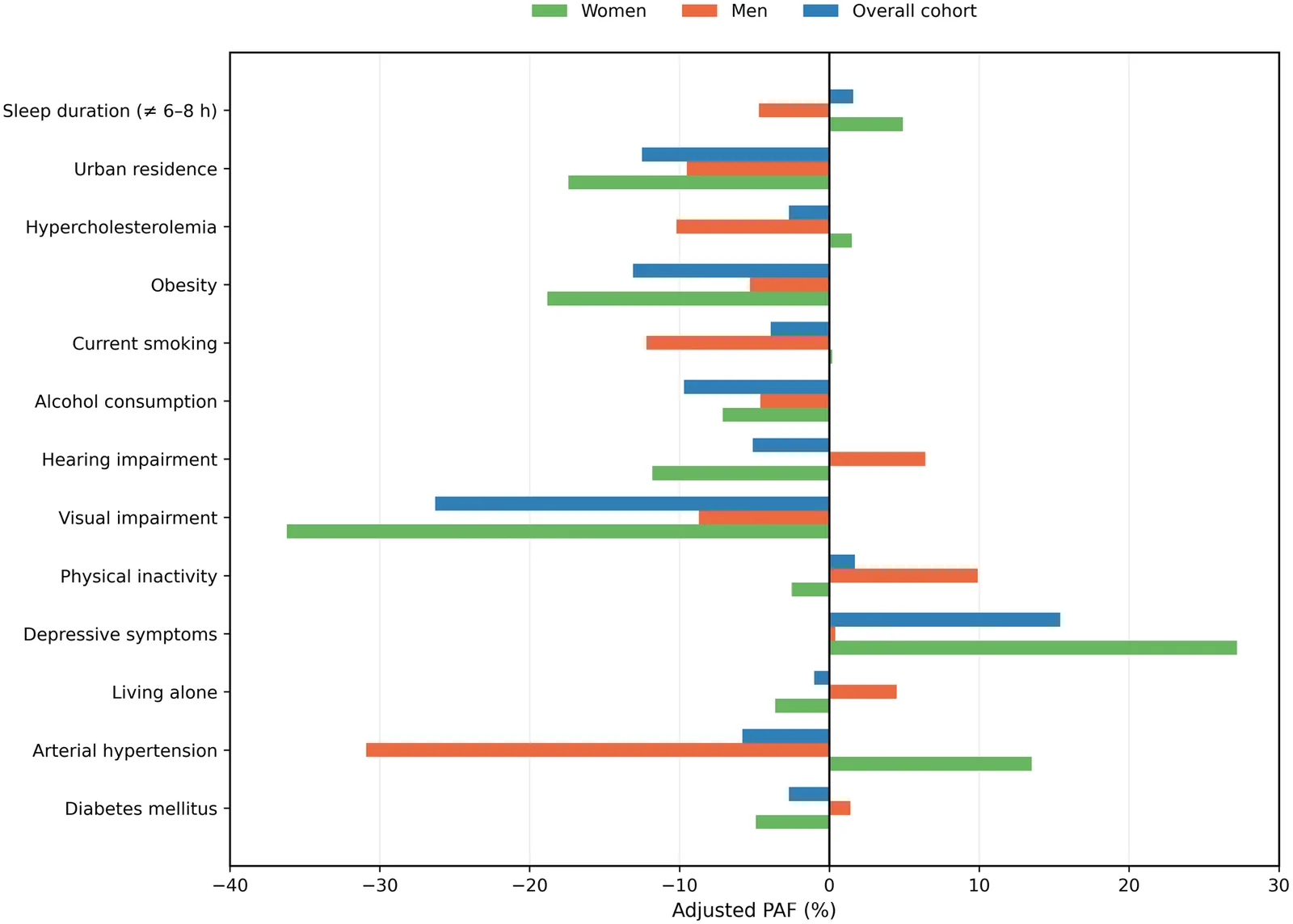
Adjusted population attributable fractions for AD-coded mortality. Bars show adjusted population attributable fractions (PAFs; point estimates) for AD-coded death (underlying cause of death) from multivariable Fine–Gray models with death from other causes as a competing event, stratified by sex and shown for the overall cohort. Positive values indicate risk-increasing associations; negative values indicate inverse associations in this competing-risk mortality framework. PAF 95% confidence intervals are reported in Table 3.

**Fig. 5 |:**
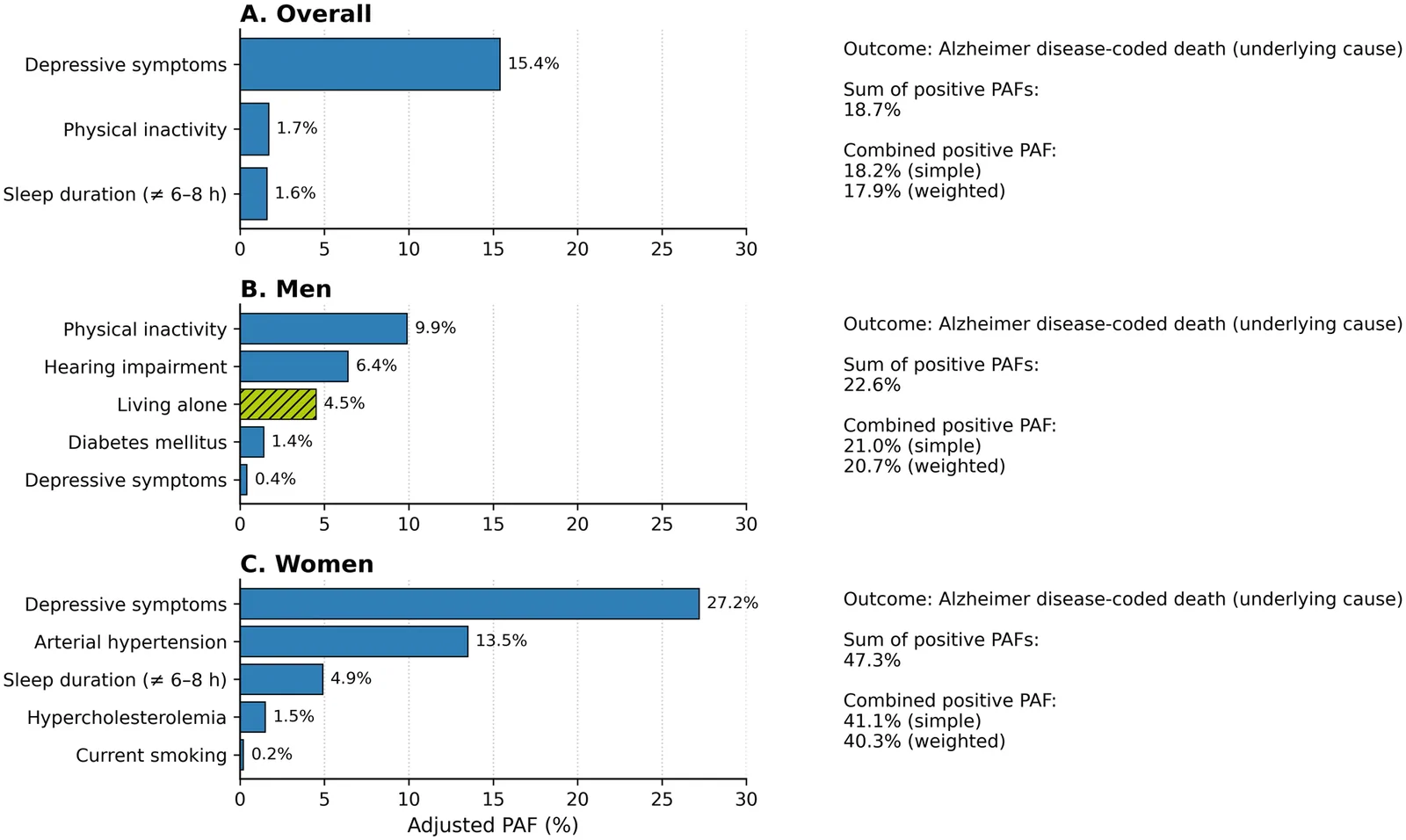
Positive population attributable fractions (PAFs) for Alzheimer disease–coded mortality and combined positive PAFs by sex. Panels show **A** the overall cohort, **B** men, and **C** women. Horizontal bars display adjusted PAFs (%) for modifiable study-entry factors with positive PAFs only (i.e., factors associated with a higher cumulative incidence of Alzheimer disease–coded death as the underlying cause of death) estimated from multivariable Fine–Gray competing-risk models with death from other causes treated as the competing event. Bars are ordered from largest to smallest within each panel. The psychosocial factor “living alone” (a proxy for social isolation) is highlighted in green to distinguish it from the remaining factors, which represent cardiometabolic/behavioral/health-related exposures. A summary box in each panel reports: (i) the sum of positive PAFs (ΣPAF^+^) and (ii) the combined positive PAF (simple and communality-weighted). Importantly, the combined positive PAF is not the arithmetic sum of the individual bars. It is calculated to avoid double-counting overlap among exposures as: $\text{Combined}\;\text{positive}\;\text{PAF}=1-\prod_{j=1}^{k}\;\left(1-\right.\text{Adjusted}\left.{\text{PAF}}_{j}\right)$. where the product is taken only over factors with positive PAFs in that panel. Individual PAF 95% confidence intervals are reported in Table 3.

Figures 4 and 5A–C summarize the adjusted PAFs: depressive symptoms produced the largest positive PAFs (particularly in women), while several factors showed negative PAFs consistent with inverse associations in this competing-risk mortality framework. When considering jointly only risk-increasing factors (combined PAFs based on positive individual PAFs), the combined PAF for AD-coded mortality was 18.2% overall (weighted 17.9%). The combined PAF was higher in women (41.1%; weighted 40.3%) than in men (21.0%; weighted 20.7%), reflecting the dominant contribution of depressive symptoms among women.

In the corresponding sensitivity analysis retaining low education and excluding urban residence (Table 5), depressive symptoms remained positively associated with AD-coded mortality in women, although the estimate was attenuated and less precise (SHR = 1.523, 95% CI 0.893–2.598; adjusted PAF = 13.7%, 95% CI − 3.4 to 32.7).

## Discussion

In this population-based cohort of older adults followed for up to 23 years, the aggregate population-level contribution of study-entry late-life modifiable factors to dementia-coded mortality was modest when estimated using a combined PAF restricted to risk-increasing factors. Within this competing-risk mortality framework, depressive symptoms at study entry emerged as the principal risk-increasing factor, particularly for AD-coded mortality and especially among women. In contrast, several cardiometabolic and sensory exposures showed inverse associations with dementia- and AD-coded deaths (SHR < 1 and negative PAFs), patterns that are plausibly shaped by reverse causation, survival selection, differential cause-of-death attribution, and competing mortality rather than biological protection.

A key implication of our findings is interpretive: in a competing-risk framework, an exposure that increases the hazard of death from other causes can reduce the cumulative incidence of dementia-coded death. Accordingly, SHRs below 1 for exposures such as smoking, obesity, arterial hypertension, or visual impairment should not be interpreted as protective against dementia neuropathology. Rather, they indicate that exposed individuals are less likely to survive long enough for dementia or AD to be recorded as the underlying cause of death, or that dementia may be differentially under-recognized or under-certified in those with substantial competing morbidity. These considerations argue against translating inverse associations with dementia mortality into prevention recommendations and underscore that late-life exposure associations with mortality are not equivalent to etiologic effects on incident dementia.

For dementia-coded mortality, the combined positive PAF was 10.8% in the total cohort (weighted, 10.7%). Although the point estimate was higher in men (32.4%; weighted, 32.2%) than in women (22.4%; weighted, 21.9%), the male estimate was driven by several individually non-significant positive PAFs and should therefore be interpreted cautiously. In women, depressive symptoms were the only statistically significant risk-increasing factor and accounted for the largest individual positive PAF (13.6%). For AD-coded mortality, the combined positive PAF was larger (18.2% overall; weighted, 17.9%) and was most pronounced in women (41.1%; weighted, 40.3%), largely reflecting the contribution of depressive symptoms.

We restricted the combined PAFs to factors with positive individual PAFs to provide an interpretable estimate of the potentially preventable fraction attributable to exposures associated with higher cumulative incidence of dementia or AD-coded death. Including negative PAFs in a single “net” combined estimate can be misleading in this setting, because inverse associations may reflect increased competing mortality or differential death-certificate attribution rather than protective effects on dementia biology. The close similarity between the weighted and unweighted combined PAFs indicates that, in our data, accounting for non-independence among included risk-increasing factors through communality-based weighting had little impact—consistent with generally low communalities for these factors.

In sensitivity analyses that retained education and excluded urban residence (Tables 4 and 5), key associations were generally consistent in direction, although effect estimates for depressive symptoms among women were attenuated and less precise. Low education showed inverse associations with dementia- and AD-coded mortality, which may reflect differential certification of dementia/AD as the underlying cause of death and/or competing mortality in socioeconomically disadvantaged groups. These findings underscore that socioeconomic measures can be highly correlated and that attributable-fraction estimates can be sensitive to how these factors are modeled.

Depressive symptoms in late life emerged as the most consistently observed risk-increasing factor for dementia- and AD-coded mortality in the primary analyses, with the clearest evidence for AD-coded mortality among women. In Table 3, depressive symptoms were associated with substantially higher AD-coded mortality in the overall cohort (SHR 1.76) and among women (SHR 2.23), with corresponding positive PAFs whose 95% CIs excluded 0 in both analyses. These observations align with longitudinal studies linking depression and depressive symptoms to incident dementia, cognitive decline, and worse outcomes among individuals with cognitive impairment^8–10,43–45^. Our findings extend this literature to a mortality endpoint and suggest that late-life depressive symptoms may be associated with a higher probability of AD being recorded as the underlying cause of death—potentially through mechanisms related to accelerated cognitive decline, reduced cognitive/brain reserve, poorer general health, reduced engagement with preventive care, or adverse vascular and inflammatory pathways. Regardless of mechanism, the sex-specific pattern is clinically relevant given the higher prevalence of depressive symptoms among women at study entry (Table 1) and the larger depressive symptoms-attributable fraction of AD-coded mortality in women.

In the Reasons for Geographic and Racial Differences in Stroke (REGARDS) cohort, Sawyer et al.^46^ examined circulating biomarkers of neurodegeneration—including glial fibrillary acidic protein, neurofilament light chain, total tau, and ubiquitin carboxy-terminal hydrolase L1—in relation to long-term all-cause and dementia-specific mortality in a racially diverse U.S. sample. Using survival models that accounted for competing risks, they found that higher baseline concentrations, particularly of glial fibrillary acidic protein and neurofilament light chain, were associated with substantially increased risk of dementia-specific mortality and were also related to all-cause (and, in some analyses, cardiovascular) mortality^46^.

Although REGARDS^46^ differs from NEDICES in its exposure domain and outcome definition (biomarkers vs modifiable risk factors; dementia-specific mortality adjudication vs dementia/AD-coded underlying cause of death), both studies converge on a key interpretive point: in older populations, dementia mortality must be analyzed in the context of substantial competing mortality, and estimates can shift depending on whether the goal is etiologic inference or prediction of cause-of-death outcomes. Taken together, these data support a framework in which upstream neurodegeneration-related biology (captured by blood biomarkers) and potentially modifiable psychosocial factors (such as depressive symptoms) jointly shape long-term dementia/AD mortality patterns, while underscoring the importance of transparent endpoint definition and careful interpretation when comparing cohorts and translating findings into prevention priorities.

We observed inverse associations of obesity with dementia-coded mortality (overall SHR 0.57; women SHR 0.50) and with AD-coded mortality (overall SHR 0.58; women SHR 0.52), as well as a strong inverse association of arterial hypertension with dementia-coded mortality in men (SHR 0.30) and a borderline inverse trend for AD-coded mortality in men (SHR 0.46). These patterns align with well-described “late-life paradoxes” in dementia epidemiology, where obesity and arterial hypertension—established risk factors for dementia when assessed in midlife—can show attenuated, null, or inverse associations when measured in late life^18,19,47^. Such patterns may reflect reverse causation (e.g., prodromal weight loss and blood-pressure decline preceding dementia diagnosis), survivor bias (selective survival of more resilient individuals with these exposures), and, in a cause-of-death framework, competing mortality that reduces the probability of living long enough for dementia to be coded as the underlying cause^20–22^. Our findings reinforce the importance of considering the timing of exposure assessment and distinguishing between risk factors for incident dementia and factors associated with dementia-coded death.

The inverse association between current smoking and dementia-coded mortality in the overall analysis is consistent with competing mortality: smokers experience higher mortality from other causes and therefore may be less likely to survive to a point at which dementia is recorded as the underlying cause of death^17^. In this context, an inverse SHR (and negative PAF) should be interpreted as a marker of competing-risk dynamics rather than evidence that smoking reduces dementia risk.

An unexpected finding was the inverse association between self-reported visual impairment and AD-coded mortality, including a large negative PAF in women. In light of dementia-prevention frameworks that include sensory impairment as a modifiable contributor to dementia incidence^8^, a genuinely protective effect of vision impairment on AD mortality is unlikely. More plausible explanations include: (i) competing mortality and frailty associated with severe sensory impairment; and/or (ii) diagnostic and certification bias, whereby dementia may be under-recognized or less likely to be recorded as the underlying cause in individuals with substantial comorbidity or disability. This finding illustrates why competing-risk methods and careful clinical interpretation are essential when evaluating late-life risk factors against a mortality endpoint.

Sleep duration outside 6–8 h was not clearly associated with dementia- or AD-coded mortality in our analyses. This differs from earlier work in this cohort, in which long sleep duration predicted dementia-related mortality over 13 years^16^. This discrepancy may reflect differences in exposure definition—our dichotomous category pooled short and long sleepers and did not isolate extreme long sleep (e.g., >10 h/night)—as well as the longer follow-up horizon and the influence of reverse causation in late life^16^. Overall, evidence linking sleep to dementia-specific mortality remains limited, and future studies with repeated and objective sleep measures are needed to clarify directionality and specificity^48–50^.

A potential concern is that inverse associations may reflect bias; however, this does not diminish the value of competing-risk analysis. On the contrary, competing-risk methods are essential because they estimate the cumulative incidence of dementia/AD-coded death in the real world, where many older adults die from other causes first^20–22^. This framework makes explicit that an exposure can increase dementia incidence while simultaneously increasing competing mortality, producing inverse associations when dementia-coded death is the endpoint. By quantifying individual PAFs (with CIs) and combined PAFs restricted to risk-increasing factors, we provide an interpretable prioritization of late-life targets associated with a higher probability of dementia/AD being recorded as the underlying cause of death—while avoiding the misleading inference that factors with negative PAFs are “protective.”

The combined PAF for dementia-coded mortality in our cohort (~11% overall) is smaller than combined PAFs often reported for incident dementia^8–11^, which is expected given: (i) late-life exposure assessment (susceptible to reverse causation and selection); (ii) our mortality endpoint (underlying cause of death) which is downstream of both dementia incidence and survival; and (iii) imperfect dementia/AD certification on death certificates^4–6^. Importantly, our combined PAFs were computed only from risk-increasing factors and are therefore not directly comparable to “all-factor” combined PAFs for incident dementia that assume causal, independent, upstream effects across the life course.

These findings refine the prevention narrative for dementia-coded mortality in three ways. First, they support systematic detection and management of depressive symptoms in older adults as a plausible strategy to reduce AD-coded mortality, particularly among women^8–11^. Second, they caution against simplistic interpretations of inverse late-life associations between obesity or arterial hypertension and dementia outcomes; these patterns likely reflect reverse causation, selection, competing mortality, and/or certification dynamics rather than true protection^18–22^. Third, they reinforce the importance of a life-course approach, whereby midlife risk-factor reduction remains the most plausible pathway for lowering dementia incidence and the downstream probability that dementia appears as the underlying cause of death^8–11^.

Strengths of this study include its population-based design, long follow-up, and explicit modeling of competing mortality using Fine–Gray subdistribution hazards, consistent with recommendations for cause-of-death endpoints in older adults^20–22^. We quantified both individual and combined PAFs and reported correlation-weighted estimates, facilitating comparison with established prevention frameworks^11^. In addition, reporting PAF confidence intervals clarifies which attributable fractions are compatible with chance variation.

Generalizability of these findings should be considered in light of the cohort’s design and the outcome definition. NEDICES is a population-based, community-dwelling cohort drawn from three socioeconomically and geographically distinct areas of central Spain, which supports internal validity for older adults living in similar European settings. However, exposures were assessed at study entry in late life (≥65 years), and the outcome was dementia- or AD-coded death as the underlying cause on death certificates; therefore, our estimates may not generalize to (i) younger populations, (ii) cohorts with substantially different life-course risk profiles, healthcare access, or certification practices, or (iii) studies using incident dementia as the outcome. In addition, our complete-case analytic sample excluded 1805 participants with missing study-entry covariates and was somewhat younger, more educated, and included fewer women than excluded participants, which may limit external validity and could influence the magnitude of sex-specific attributable fractions. These considerations suggest that the primary inferences—particularly the prominence of depressive symptoms among women in this competing-risk mortality framework—are most applicable to older community populations with comparable demographic structure and mortality ascertainment, and should be confirmed in other cohorts and settings.

Several limitations merit consideration. First, exposures were assessed at study entry in late life; this timing likely reflects risk profiles after substantial subclinical neurodegeneration and/or vascular disease processes may already be underway, increasing the risk of reverse causation and survivor bias^17–19^. Second, several exposures were self-reported and measured only once, which may introduce misclassification and attenuate or distort associations. Third, dietary quality measures were unavailable at study entry, preventing evaluation of diet-related prevention targets. Fourth, cause-of-death attribution is imperfect: dementia and AD are often omitted as the underlying cause of death^4–6^, leading to outcome misclassification that may be non-differential (biasing associations toward the null) or differential (e.g., competing cardiometabolic causes being preferentially listed among those with high vascular burden), potentially contributing to inverse associations. Fifth, complete-case analysis yielded a sample that was somewhat younger and more educated than excluded participants, with fewer women, raising the possibility of selection bias. This pattern of missingness is particularly relevant to the sex-specific findings because depressive symptoms were more prevalent among women at study entry. If excluded women were older, frailer, or differed in depressive symptom burden and competing mortality risk, the magnitude of female-specific SHRs and PAFs for depressive symptoms (which depend on both exposure prevalence and the adjusted association) could be under- or over-estimated. We therefore interpret sex-specific PAF magnitudes cautiously. In the primary models, depressive symptoms were the largest risk-increasing contributor for AD-coded mortality among women; however, in sensitivity analyses retaining education (and excluding urban residence), associations for depressive symptoms were attenuated and less precise, suggesting partial confounding or shared variance with socioeconomic factors (Tables 4 and 5). Finally, sex-specific analyses of AD-coded mortality in men were limited by fewer events, resulting in wider confidence intervals and possible type II error.

In closing, in this large, population-based cohort with more than two decades of follow-up, depressive symptoms at study entry emerged as the most consistent risk-increasing correlate of dementia- and AD-coded mortality. The association with dementia-coded mortality was statistically significant only among women, whereas depressive symptoms were associated with higher AD-coded mortality both in the overall cohort and, more strongly, among women. In women, depressive symptoms were associated with more than twice the subdistribution hazard of AD-coded death and yielded the largest positive PAF, contributing substantially to the combined positive PAF of 41.1% for AD-coded mortality. These findings should nevertheless be interpreted cautiously because the associations were attenuated in sensitivity analyses incorporating educational attainment, exposures were measured only once in late life, and the outcomes reflected dementia or AD recorded as the underlying cause of death rather than incident disease. In contrast, several cardiometabolic and sensory factors showed inverse associations that are more plausibly explained by reverse causation, survivor selection, competing mortality, and differential death-certificate attribution than by genuine biological protection. Overall, the findings support systematic recognition and management of depressive symptoms in older adults, particularly women, while reinforcing the need for life-course prevention strategies and replication in independent cohorts.

## Figures and Tables

**Table 1 |:** Study-entry demographic and modifiable risk factor characteristics stratified by sex (*N* = 3085)

Characteristic	Men (*N* = 1426)	Women (*N* = 1659)	Test statistics	*P* value
Age, mean ± SD, years	73.1 ± 6.2	73.6 ± 6.5	*t* = −2.00	0.046*
Low education	1164 (81.6%)	1477 (89.0%)	χ^2^ = 34.10	5.217 × 10^−9^*
Urban residence	813 (57.0%)	992 (59.8%)	χ^2^ = 2.446	0.118
Current smoking	312 (21.9%)	57 (3.4%)	χ^2^ = 247.72	8.170 × 10^−56^*
Alcohol consumption	721 (50.6%)	333 (20.1%)	χ^2^ = 316.92	6.790 × 10^−71^*
Diabetes mellitus	223 (15.6%)	287 (17.3%)	χ^2^ = 1.534	0.216
Arterial hypertension	628 (44.0%)	956 (57.6%)	χ^2^ = 56.66	5.188 × 10^−14^*
Hypercholesterolemia	344 (24.1%)	565 (34.1%)	χ^2^ = 36.41	1.602 × 10^−9^*
Depressive symptoms	228 (16.0%)	505 (30.4%)	χ^2^ = 88.41	5.326 × 10^−21^*
Obesity	308 (21.6%)	543 (32.7%)	χ^2^ = 47.57	5.308 × 10^−12^*
Physical inactivity	426 (29.9%)	346 (20.9%)	χ^2^ = 33.24	8.155 × 10^−9^*
Visual impairment	711 (49.8%)	951 (57.3%)	χ^2^ = 17.19	3.379 × 10^−5^*
Hearing impairment	424 (29.7%)	498 (30.0%)	χ^2^ = 0.03	0.863
Sleep duration (≠ 6–8 h)	755 (52.9%)	854 (51.5%)	χ^2^ = 0.663	0.416
Living alone	95 (6.7%)	404 (24.4%)	χ^2^ = 176.99	2.201 × 10^−40^*

All values are n (%) unless otherwise indicated. Differences between men and women were assessed using two-sided independent-samples t tests for continuous variables and two-sided Pearson chi-square tests for categorical variables. Test statistics are reported as t or χ^2^, as appropriate. P values are asymptotic, two-sided, and unadjusted. Statistically significant differences are indicated by an asterisk (*p < 0.05). *SD* standard deviation.

**Table 2 |:** Fine–Gray subdistribution hazard ratios, adjusted population attributable fractions, prevalence, and communality for dementia-coded mortality (underlying cause of death), overall and by sex

Risk factor	SHR (95% confidence interval)	Prevalence	Communality	Adjusted population attributable fraction (%) (95% confidence interval)
Overall cohort (*n* = 3085)				
Diabetes mellitus	1.054 (0.681–1.632)	0.165	0.0151	0.9 (−5.6 to 9.4)
Arterial hypertension	0.831 (0.604–1.142)	0.513	0.0408	−9.5 (−25.5 to 6.8)
Living alone	1.072 (0.692–1.659)	0.162	0.0152	1.1 (−5.3 to 9.6)
Depressive symptoms	1.414 (0.985–2.030)	0.238	0.0146	9.0 (−0.4 to 19.7)
Physical inactivity	0.897 (0.585–1.376)	0.250	0.0014	−2.6 (−11.6 to 8.6)
Visual impairment	0.777 (0.558–1.082)	0.539	0.1084	−13.7 (−31.3 to 4.2)
Hearing impairment	0.912 (0.637–1.307)	0.299	0.0008	−2.7 (−12.2 to 8.4)
Alcohol consumption	0.729 (0.515–1.031)	0.342	0.0748	−10.2 (−19.9 to 1.0)
Current smoking	0.614 (0.412–0.914)	0.120	0.0322	−4.9 (−7.6 to −1.0)
Obesity	0.566 (0.376–0.852)	0.276	0.1539	−13.6 (−20.8 to −4.3)
Hypercholesterolemia	0.958 (0.675–1.361)	0.295	0.0000	−1.2 (−10.6 to 9.6)
Urban residence	0.973 (0.678–1.398)	0.585	0.3075	−1.6 (−23.2 to 18.9)
Sleep duration (≠ 6–8 h)	0.828 (0.587–1.168)	0.522	0.0157	−9.8 (−27.3 to 8.0)
Men (*n* = 1426)				
Diabetes mellitus	1.609 (0.762–3.397)	0.156	0.0009	8.7 (−3.9 to 27.2)
Arterial hypertension	0.296 (0.140–0.628)	0.440	0.0005	−44.9 (−60.9 to −19.6)
Living alone	1.242 (0.371–4.152)	0.067	0.0023	1.6 (−4.4 to 17.4)
Depressive symptoms	1.009 (0.449–2.266)	0.160	0.0527	0.1 (−9.7 to 16.8)
Physical inactivity	1.355 (0.663–2.771)	0.299	0.0221	9.6 (−11.2 to 34.6)
Visual impairment	0.835 (0.472–1.478)	0.499	0.2022	−9.0 (−35.8 to 19.3)
Hearing impairment	1.204 (0.667–2.175)	0.297	0.0057	5.7 (−11.0 to 25.9)
Alcohol consumption	1.259 (0.588–2.695)	0.506	0.0013	11.6 (−26.3 to 46.2)
Current smoking	0.593 (0.309–1.135)	0.219	0.0036	−9.8 (−17.8 to 2.9)
Obesity	0.819 (0.373–1.798)	0.216	0.0823	−4.1 (−15.7 to 14.7)
Hypercholesterolemia	0.628 (0.277–1.426)	0.241	0.0005	−9.8 (−21.1 to 9.3)
Urban residence	0.940 (0.498–1.774)	0.570	0.3152	−3.6 (−40.1 to 30.6)
Sleep duration (≠ 6–8 h)	0.698 (0.360–1.353)	0.529	0.0673	−19.0 (−51.2 to 15.7)
Women (*n* = 1659)				
Diabetes mellitus	0.879 (0.516–1.497)	0.173	0.0383	−2.1 (−9.1 to 7.9)
Arterial hypertension	1.141 (0.766–1.699)	0.576	0.0656	7.5 (−15.6 to 28.7)
Living alone	1.046 (0.648–1.689)	0.244	0.0426	1.1 (−9.4 to 14.4)
Depressive symptoms	1.519 (1.001–2.304)	0.304	0.0075	13.6 (0.0 to 28.4)
Physical inactivity	0.753 (0.429–1.321)	0.209	0.0016	−5.4 (−13.6 to 6.3)
Visual impairment	0.776 (0.516–1.167)	0.573	0.0492	−14.7 (−38.4 to 8.7)
Hearing impairment	0.817 (0.523–1.276)	0.300	0.0000	−5.8 (−16.7 to 7.6)
Alcohol consumption	0.670 (0.425–1.057)	0.201	0.1288	−7.1 (−13.1 to 1.1)
Current smoking	1.028 (0.488–2.165)	0.034	0.0882	0.1 (−1.8 to 3.8)
Obesity	0.496 (0.309–0.796)	0.327	0.1430	−19.7 (−29.2 to −7.1)
Hypercholesterolemia	1.054 (0.703–1.582)	0.341	0.0022	1.8 (−11.3 to 16.6)
Urban residence	0.886 (0.561–1.398)	0.598	0.2635	−7.3 (−35.6 to 19.2)
Sleep duration (≠ 6–8 h)	0.873 (0.581–1.314)	0.515	0.0045	−6.9 (−27.3 to 13.9)

*SHR* subdistribution hazard ratio estimated using Fine–Gray competing-risk regression (cumulative incidence function), with deaths from other causes as competing events; the adjusted population attributable fraction (%) (PAF) was calculated from the SHR and exposure prevalence; PAF 95% confidence intervals were obtained by applying the PAF function to the lower and upper limits of the SHR 95% confidence interval; Prevalence = study-entry proportion with the exposure (overall and by sex); Communality = PCA-derived communality from the polychoric correlation matrix used to compute the weighted combined PAF. Models for the total cohort were adjusted for age and sex; sex-stratified models were adjusted for age.

**Table 3 |:** Fine–Gray subdistribution hazard ratios, adjusted population attributable fractions, prevalence, and communality for Alzheimer disease–coded mortality (underlying cause of death), overall and by sex

Risk factor	SHR (95% confidence interval)	Prevalence	Communality	Adjusted population attributable fraction (%) (95% confidence interval)
Overall cohort (*n* = 3085)				
Diabetes mellitus	0.841 (0.460–1.540)	0.165	0.0151	−2.7 (−9.8 to 8.2)
Arterial hypertension	0.893 (0.593–1.344)	0.513	0.0408	−5.8 (−26.4 to 15.0)
Living alone	0.939 (0.510–1.729)	0.162	0.0152	−1.0 (−8.6 to 10.6)
Depressive symptoms	1.763 (1.114–2.791)	0.238	0.0146	15.4 (2.6 to 29.9)
Physical inactivity	1.070 (0.636–1.802)	0.250	0.0014	1.7 (−10.0 to 16.7)
Visual impairment	0.614 (0.398–0.947)	0.539	0.1084	−26.3 (−48.0 to −2.9)
Hearing impairment	0.836 (0.519–1.349)	0.299	0.0008	−5.1 (−16.8 to 9.4)
Alcohol consumption	0.742 (0.469–1.172)	0.342	0.0748	−9.7 (−22.2 to 5.6)
Current smoking	0.688 (0.410–1.154)	0.120	0.0322	−3.9 (−7.6 to 1.8)
Obesity	0.579 (0.345–0.974)	0.276	0.1539	−13.1 (−22.1 to −0.7)
Hypercholesterolemia	0.911 (0.572–1.451)	0.295	0.0000	−2.7 (−14.5 to 11.7)
Urban residence	0.810 (0.513–1.279)	0.585	0.3075	−12.5 (−39.8 to 14.0)
Sleep duration (≠ 6–8 h)	1.032 (0.656–1.622)	0.522	0.0157	1.6 (−21.8 to 24.4)
Men (*n* = 1426)				
Diabetes mellitus	1.093 (0.402–2.977)	0.156	0.0009	1.4 (−10.3 to 23.6)
Arterial hypertension	0.464 (0.215–1.000)	0.440	0.0005	−30.9 (−52.8 to 0.0)
Living alone	1.703 (0.501–5.789)	0.067	0.0023	4.5 (−3.5 to 24.3)
Depressive symptoms	1.024 (0.382–2.744)	0.160	0.0527	0.4 (−11.0 to 21.8)
Physical inactivity	1.368 (0.627–2.986)	0.299	0.0221	9.9 (−12.6 to 37.3)
Visual impairment	0.840 (0.447–1.578)	0.499	0.2022	−8.7 (−38.1 to 22.4)
Hearing impairment	1.231 (0.625–2.427)	0.297	0.0057	6.4 (−12.5 to 29.8)
Alcohol consumption	0.914 (0.411–2.031)	0.506	0.0013	−4.6 (−42.5 to 34.3)
Current smoking	0.502 (0.243–1.036)	0.219	0.0036	−12.2 (−19.9 to 0.8)
Obesity	0.769 (0.312–1.895)	0.216	0.0823	−5.3 (−17.5 to 16.2)
Hypercholesterolemia	0.616 (0.235–1.614)	0.241	0.0005	−10.2 (−22.6 to 12.9)
Urban residence	0.847 (0.402–1.785)	0.570	0.3152	−9.5 (−51.7 to 30.9)
Sleep duration (≠ 6–8 h)	0.915 (0.427–1.962)	0.529	0.0673	−4.7 (−43.5 to 33.7)
Women (*n* = 1659)				
Diabetes mellitus	0.731 (0.342–1.562)	0.173	0.0383	−4.9 (−12.8 to 8.9)
Arterial hypertension	1.270 (0.730–2.208)	0.576	0.0656	13.5 (−18.4 to 41.0)
Living alone	0.857 (0.425–1.730)	0.244	0.0426	−3.6 (−16.3 to 15.1)
Depressive symptoms	2.228 (1.278–3.883)	0.304	0.0075	27.2 (7.8 to 46.7)
Physical inactivity	0.884 (0.426–1.835)	0.209	0.0016	−2.5 (−13.6 to 14.9)
Visual impairment	0.536 (0.300–0.956)	0.573	0.0492	−36.2 (−67.0 to −2.6)
Hearing impairment	0.649 (0.338–1.244)	0.300	0.0000	−11.8 (−24.8 to 6.8)
Alcohol consumption	0.671 (0.357–1.259)	0.201	0.1288	−7.1 (−14.8 to 4.9)
Current smoking	1.060 (0.369–3.050)	0.034	0.0882	0.2 (−2.2 to 6.5)
Obesity	0.517 (0.276–0.967)	0.327	0.1430	−18.8 (−31.0 to −1.1)
Hypercholesterolemia	1.046 (0.598–1.828)	0.341	0.0022	1.5 (−15.9 to 22.0)
Urban residence	0.753 (0.414–1.367)	0.598	0.2635	−17.4 (−53.9 to 18.0)
Sleep duration (≠ 6–8 h)	1.101 (0.625–1.941)	0.515	0.0045	4.9 (−23.8 to 32.5)

*SHR* subdistribution hazard ratio estimated using Fine–Gray competing-risk regression (cumulative incidence function), with deaths from other causes as competing events, the adjusted population attributable fraction (%) (PAF) was calculated from the SHR and exposure prevalence, *PAF* 95% confidence intervals were obtained by applying the PAF function to the lower and upper limits of the SHR 95% confidence interval, Prevalence = study-entry proportion with the exposure (overall and by sex), Communality = *PCA*-derived communality from the polychoric correlation matrix used to compute the weighted combined PAF. Models for the total cohort were adjusted for age and sex; sex-stratified models were adjusted for age.

**Table 4 |:** Fine–Gray subdistribution hazard ratios, adjusted population attributable fractions, prevalence, and communality for dementia-coded mortality (underlying cause of death), overall and by sex, including the low educational level factor and removing the urban residence factor

Risk factor	SHR (95% confidence interval)	Prevalence	Communality	Adjusted population attributable fraction (%) (95% confidence interval)
Overall cohort (*n* = 3085)				
Low educational level	0.631 (0.427–0.934)	0.856	0.4295	−46.1 (−96.4 to −5.9)
Diabetes mellitus	1.056 (0.711–1.569)	0.165	0.4480	0.9 (−5.0 to 8.6)
Arterial hypertension	0.822 (0.615–1.100)	0.513	0.5229	−10.0 (−24.6 to 4.9)
Living alone	1.103 (0.757–1.607)	0.162	0.4194	1.6 (−4.1 to 9.0)
Depressive symptoms	1.189 (0.854–1.656)	0.238	0.4267	4.3 (−3.6 to 13.5)
Physical inactivity	1.135 (0.780–1.651)	0.250	0.8150	3.3 (−5.8 to 14.0)
Visual impairment	0.788 (0.588–1.057)	0.539	0.5097	−12.9 (−28.5 to 3.0)
Hearing impairment	0.855 (0.614–1.190)	0.299	0.1416	−4.5 (−13.0 to 5.4)
Alcohol consumption	0.729 (0.535–0.994)	0.342	0.6873	−10.2 (−18.9 to −0.2)
Current smoking	0.558 (0.391–0.798)	0.120	0.7932	−5.6 (−7.9 to −2.5)
Obesity	0.643 (0.443–0.934)	0.276	0.4406	−10.9 (−18.2 to −1.9)
Hypercholesterolemia	0.983 (0.716–1.350)	0.295	0.3464	−0.5 (−9.1 to 9.3)
Sleep duration (≠ 6–8 h)	0.842 (0.612–1.159)	0.522	0.7704	−8.9 (−25.3 to 7.6)
Men (*n* = 1426)				
Low educational level	0.716 (0.377–1.359)	0.816	0.4574	−30.1 (−103.3 to 22.7)
Diabetes mellitus	1.435 (0.742–2.772)	0.156	0.4221	6.3 (−4.2 to 21.7)
Arterial hypertension	0.450 (0.255–0.794)	0.440	0.6124	−31.9 (−48.7 to −10.0)
Living alone	1.133 (0.401–3.200)	0.067	0.6518	0.9 (−4.2 to 12.8)
Depressive symptoms	1.119 (0.573–2.185)	0.160	0.3364	1.9 (−7.3 to 15.9)
Physical inactivity	1.726 (0.931–3.199)	0.299	0.8138	17.8 (−2.1 to 39.7)
Visual impairment	0.801 (0.480–1.338)	0.499	0.6374	−11.0 (−35.1 to 14.4)
Hearing impairment	1.080 (0.633–1.842)	0.297	0.5658	2.3 (−12.2 to 20.0)
Alcohol consumption	1.306 (0.668–2.550)	0.506	0.7082	13.4 (−20.2 to 44.0)
Current smoking	0.510 (0.295–0.882)	0.219	0.6654	−12.0 (−18.3 to −2.7)
Obesity	0.883 (0.440–1.771)	0.216	0.5576	−2.6 (−13.8 to 14.3)
Hypercholesterolemia	0.645 (0.315–1.321)	0.241	0.3356	−9.4 (−19.8 to 7.2)
Sleep duration (≠ 6–8 h)	0.670 (0.369–1.217)	0.529	0.8338	−21.1 (−50.1 to 10.3)
Women (*n* = 1659)				
Low educational level	0.577 (0.352–0.946)	0.890	0.5069	−60.3 (−136.1 to −5.1)
Diabetes mellitus	0.923 (0.564–1.508)	0.173	0.4583	−1.4 (−8.2 to 8.1)
Arterial hypertension	1.026 (0.714–1.475)	0.576	0.5129	1.5 (−19.7 to 21.5)
Living alone	1.026 (0.676–1.558)	0.244	0.2914	0.6 (−8.6 to 12.0)
Depressive symptoms	1.183 (0.803–1.742)	0.304	0.4820	5.3 (−6.4 to 18.4)
Physical inactivity	0.959 (0.589–1.562)	0.209	0.8141	−0.9 (−9.4 to 10.5)
Visual impairment	0.783 (0.545–1.124)	0.573	0.3503	−14.2 (−35.3 to 6.6)
Hearing impairment	0.783 (0.516–1.188)	0.300	0.4289	−7.0 (−17.0 to 5.3)
Alcohol consumption	0.650 (0.434–0.972)	0.201	0.4629	−7.6 (−12.8 to −0.6)
Current smoking	0.969 (0.526–1.787)	0.034	0.6468	−0.1 (−1.6 to 2.6)
Obesity	0.582 (0.377–0.899)	0.327	0.2977	−15.8 (−25.6 to −3.4)
Hypercholesterolemia	1.075 (0.744–1.555)	0.341	0.4556	2.5 (−9.6 to 15.9)
Sleep duration (≠ 6–8 h)	0.901 (0.617–1.315)	0.515	0.7452	−5.4 (−24.4 to 13.9)

*SHR* = subdistribution hazard ratio estimated using Fine–Gray competing-risk regression (cumulative incidence function), with deaths from other causes as competing events; the adjusted population attributable fraction (%) (PAF) was calculated from the SHR and exposure prevalence, *PAF* 95% confidence intervals were obtained by applying the *PAF* function to the lower and upper limits of the SHR 95% confidence interval; Prevalence = study-entry proportion with the exposure (overall and by sex), Communality = *PCA*-derived communality from the polychoric correlation matrix used to compute the weighted combined PAF. Models for the total cohort were adjusted for age and sex, sex-stratified models were adjusted for age.

**Table 5 |:** Fine–Gray subdistribution hazard ratios, adjusted population attributable fractions, prevalence, and communality for Alzheimer disease–coded mortality (underlying cause of death), overall and by sex, including the low educational level factor and removing the urban residence factor

Risk factor	SHR (95% confidence interval)	Prevalence	Communality	Adjusted population attributable fraction (%) (95% confidence interval)
Overall cohort (*n* = 3085)				
Low educational level	0.703 (0.416–1.188)	0.856	0.4295	−34.1 (−99.8 to 13.8)
Diabetes mellitus	0.844 (0.482–1.478)	0.165	0.4480	−2.6 (−9.4 to 7.3)
Arterial hypertension	0.868 (0.595–1.266)	0.513	0.5229	−7.3 (−26.2 to 12.0)
Living alone	1.031 (0.617–1.724)	0.162	0.4194	0.5 (−6.6 to 10.5)
Depressive symptoms	1.342 (0.872–2.065)	0.238	0.4267	7.5 (−3.1 to 20.2)
Physical inactivity	1.195 (0.759–1.884)	0.250	0.8150	4.7 (−6.4 to 18.1)
Visual impairment	0.656 (0.448–0.960)	0.539	0.5097	−22.7 (−42.3 to −2.2)
Hearing impairment	0.814 (0.525–1.262)	0.299	0.1416	−5.9 (−16.6 to 7.3)
Alcohol consumption	0.706 (0.469–1.061)	0.342	0.6873	−11.2 (−22.2 to 2.0)
Current smoking	0.664 (0.424–1.040)	0.120	0.7932	−4.2 (−7.4 to 0.5)
Obesity	0.643 (0.397–1.040)	0.276	0.4406	−10.9 (−20.0 to 1.1)
Hypercholesterolemia	0.905 (0.589–1.391)	0.295	0.3464	−2.9 (−13.8 to 10.3)
Sleep duration (≠ 6–8 h)	1.184 (0.780–1.797)	0.522	0.7704	8.7 (−12.9 to 29.3)
Men (*n* = 1426)				
Low educational level	0.727 (0.338–1.563)	0.816	0.4574	−28.7 (−117.5 to 31.5)
Diabetes mellitus	0.816 (0.310–2.145)	0.156	0.4221	−3.0 (−12.1 to 15.2)
Arterial hypertension	0.552 (0.289–1.054)	0.440	0.6124	−24.6 (−45.6 to 2.3)
Living alone	1.594 (0.564–4.503)	0.067	0.6518	3.8 (−3.0 to 19.0)
Depressive symptoms	1.098 (0.485–2.486)	0.160	0.3364	1.5 (−9.0 to 19.2)
Physical inactivity	1.699 (0.860–3.355)	0.299	0.8138	17.3 (−4.4 to 41.3)
Visual impairment	0.768 (0.426–1.383)	0.499	0.6374	−13.1 (−40.1 to 16.0)
Hearing impairment	1.096 (0.589–2.040)	0.297	0.5658	2.8 (−13.9 to 23.6)
Alcohol consumption	0.897 (0.451–1.785)	0.506	0.7082	−5.5 (−38.5 to 28.4)
Current smoking	0.487 (0.259–0.916)	0.219	0.6654	−12.7 (−19.4 to −1.9)
Obesity	0.804 (0.352–1.837)	0.216	0.5576	−4.4 (−16.3 to 15.3)
Hypercholesterolemia	0.623 (0.261–1.490)	0.241	0.3356	−10.0 (−21.7 to 10.6)
Sleep duration (≠ 6–8 h)	0.965 (0.483–1.927)	0.529	0.8338	−1.9 (−37.6 to 32.9)
Women (*n* = 1659)				
Low educational level	0.701 (0.335–1.468)	0.890	0.5069	−36.3 (−145.2 to 29.4)
Diabetes mellitus	0.859 (0.425–1.735)	0.173	0.4583	−2.5 (−11.0 to 11.3)
Arterial hypertension	1.166 (0.698–1.947)	0.576	0.5129	8.7 (−21.0 to 35.3)
Living alone	0.958 (0.530–1.732)	0.244	0.2914	−1.0 (−13.0 to 15.2)
Depressive symptoms	1.523 (0.893–2.598)	0.304	0.4820	13.7 (−3.4 to 32.7)
Physical inactivity	0.898 (0.471–1.715)	0.209	0.8141	−2.2 (−12.4 to 13.0)
Visual impairment	0.624 (0.373–1.043)	0.573	0.3503	−27.5 (−56.0 to 2.4)
Hearing impairment	0.675 (0.367–1.240)	0.300	0.4289	−10.8 (−23.4 to 6.7)
Alcohol consumption	0.606 (0.337–1.088)	0.201	0.4629	−8.6 (−15.4 to 1.7)
Current smoking	0.939 (0.389–2.264)	0.034	0.6468	−0.2 (−2.1 to 4.1)
Obesity	0.596 (0.331–1.071)	0.327	0.2977	−15.2 (−28.0 to 2.3)
Hypercholesterolemia	1.070 (0.632–1.811)	0.341	0.4556	2.3 (−14.3 to 21.7)
Sleep duration (≠ 6–8 h)	1.330 (0.789–2.242)	0.515	0.7452	14.5 (−12.1 to 38.9)

*SHR* = subdistribution hazard ratio estimated using Fine–Gray competing−risk regression (cumulative incidence function), with deaths from other causes as competing events, the adjusted population attributable fraction (%) (PAF) was calculated from the SHR and exposure prevalence, PAF 95% confidence intervals were obtained by applying the PAF function to the lower and upper limits of the SHR 95% confidence interval, Prevalence = study-entry proportion with the exposure (overall and by sex), Communality = PCA-derived communality from the polychoric correlation matrix used to compute the weighted combined PAF. Models for the total cohort were adjusted for age and sex, sex-stratified models were adjusted for age.

## Data Availability

The data that support the findings of this study are derived from the Neurological Disorders in Central Spain (NEDICES) study. Due to ethical and legal restrictions related to participant confidentiality and data protection, the dataset is not publicly available. Access to de-identified data may be granted upon reasonable request to the corresponding author, subject to approval by the NEDICES Steering Committee and the relevant institutional ethics committees, and in accordance with applicable data-sharing agreements and regulations.

## Code availability

The statistical code used for the analyses was written for Stata/SE v17.0 and used the stcrreg command for Fine–Gray competing-risk models, with additional code for population attributable fraction and communality-weighted calculations. Code may be made available by the corresponding author, subject to review to ensure that no sensitive, identifiable, or data-derived confidential information from the NEDICES cohort is disclosed. Because the underlying dataset contains sensitive personal health information and is not publicly available, the code cannot be used to reproduce the analyses without approved access to the de-identified study data.
